# In what sense are dogs special? Canine cognition in comparative context

**DOI:** 10.3758/s13420-018-0349-7

**Published:** 2018-09-24

**Authors:** Stephen E. G. Lea, Britta Osthaus

**Affiliations:** 1 Department of Psychology, University of Exeter, Washington Singer Laboratories, Exeter, EX4 4QG UK; 2 School of Psychology, Politics and Sociology, Canterbury Christ Church University, Canterbury, CT1 1QU UK

**Keywords:** Dog, Cognition, Carnivoran, Social hunting, Domestic animal, Comparative cognition

## Abstract

The great increase in the study of dog cognition in the current century has yielded insights into canine cognition in a variety of domains. In this review, we seek to place our enhanced understanding of canine cognition into context. We argue that in order to assess dog cognition, we need to regard dogs from three different perspectives: phylogenetically, as carnivoran and specifically a canid; ecologically, as social, cursorial hunters; and anthropogenically, as a domestic animal. A principled understanding of canine cognition should therefore involve comparing dogs’ cognition with that of other carnivorans, other social hunters, and other domestic animals. This paper contrasts dog cognition with what is known about cognition in species that fit into these three categories, with a particular emphasis on wolves, cats, spotted hyenas, chimpanzees, dolphins, horses, and pigeons. We cover sensory cognition, physical cognition, spatial cognition, social cognition, and self-awareness. Although the comparisons are incomplete, because of the limited range of studies of some of the other relevant species, we conclude that dog cognition is influenced by the membership of all three of these groups, and taking all three groups into account, dog cognition does not look exceptional.

The present paper is not a complete review of canine cognition. Others have attempted that formidable task—for example, Bensky, Gosling, and Sinn (2013), Miklósi (2014), and Arden, Bensky, and Adams (2016)—and we are not intending to duplicate their efforts. Our aim is different: it is to set canine cognition into a comparative context, and by so doing to investigate whether the cognitive capacities of dogs are, as has been claimed in recent years, some kind of special case; or whether, instead, they are what we would expect when we put dogs alongside the appropriate comparison groups.

Dogs have been used in psychological and behavioral experiments for almost as long as such experiments have been performed: as a result of the long history of their use as “model organisms” in biomedical research, they found their way into various kinds of psychological investigation very early on. The most famous example was Pavlov’s (1927) foundational work on salivary conditioning, which expanded into an entire school of investigation in the Soviet Union and, between 1945 and 1990, the Soviet satellite states in Eastern and Central Europe (see Wyrwicka, 1994). But dogs were also put to use in the model organism phase of Western comparative psychology, for example, in avoidance learning experiments (e.g., Brush, Brush, & Solomon, 1955; Solomon & Wynne, 1953). Despite its name, however, early “comparative psychology” research did not allow for much useful comparison between species, since the species used were chosen for convenience rather than to allow comparisons motivated by any kind of evolutionary theory. We agree with the view of Kamil (1998) that an integrated account of animal cognition must take an evolutionary standpoint, and that even the most elementary and ubiquitous phenomena of animal learning, such as classical and instrumental conditioning, must be seen as the modification of behavior systems that have emerged through evolution (Timberlake, 1993, 1994). And these considerations apply as strongly to the cognition of a single species—in our case, the dog—as they do to animals in general.

Taking these arguments into account, what would be the appropriate, evolutionarily informative comparison species for the domestic dog? To put it another way, how should we approach the task of truly setting canine cognition into its comparative context? What other species should we compare dogs and their cognition with? All and any of them? That would be an impossible task; even among the vertebrates, there are more than 5,000 other species of mammal, more than 8,000 species of bird, about 28,000 species of teleost fish, and numerous members of other classes to consider; and then there are the uncountable numbers of invertebrate species. Merely taking a random selection of all these other species into a comparison with dogs would be irrational to the point of absurdity—yet that is what we would find ourselves doing if we simply looked for references to research on dogs in one of the great texts on animal cognition, such as Mackintosh’s (1974) survey of animal learning at the end of its heyday, or Shettleworth’s (2010) study of animal cognition in the context of behavior and its evolution. Why should we compare dog cognition with the cognition of pigeons, rats, or rhesus monkeys, as we would have to if we were using Mackintosh’s book, or with the behavior of chickadees, voles, and chimpanzees, as Shettleworth’s book would allow us to?

Before we can decide what are the appropriate comparisons to make, we need to decide what we should be looking for in comparing the cognition of different species. Despite a recent revival of interest in the project of placing species on a single ordering by intelligence (see, for example, Burkart, Schubiger, & Van Schaik, 2017), classically referred to as a *scala naturae* (“ladder of nature”), we are not seeking to place dogs within such a ranking. Nor, however, do we share the belief of Macphail (1987) that there are no interesting cognitive differences between any nonhuman animal species. Rather, we take the view (espoused, for example, by Kamil, 1998) that there will be cognitive differences between species, and groups of species, but to understand these we shall have to put them into the context of the ecological niche, as well as the phylogenetic position, of the species concerned. All of this does assume that it is possible to demonstrate cognitive differences between species in a way that is not confounded by sensory, motor, or motivational differences. The problems of doing so have been well rehearsed, but so have the solutions (e.g., Bitterman, 1965). An additional complication, certainly with highly social species such as dogs, is that rearing conditions and the kinds of social interactions going on in an experiment may well impact on performance in cognitive tests. For detailed comparisons it is therefore important that different species should be raised in similar ways, which has rarely been the case except in some recent comparisons between dogs and wolves (e.g., Marshall-Pescini, Schwarz, Kostelnik, Virányi, & Range, 2017), and that procedures should be as standardized, and as fully documented, as possible.

In practice, standardization of procedures across species has rarely been achieved, even where it is theoretically possible. This means that we have to take the alternative route proposed by Bitterman (1965), and focus our attention as much as possible on cognitive challenges that have been presented using multiple different methods, and with parametric variations within each method; and we need to look primarily for qualitative differences in response to cognitive challenges, rather than quantitative ones. The literature on dog cognition is now sufficiently extensive to make this a realistic program in at least some domains, but it is still developing rapidly; and for many of the species we will want to compare with dogs, we have far fewer studies. So all our conclusions, especially conclusions about a species not showing some particular cognitive capacity, need to carry the implicit caveat that future research might change our views.

Accepting that caveat, we need to proceed to decide what comparisons we should meaningfully make. To do that, we need to answer the question posed by Coppinger and Coppinger (2016) in the title of their recent book, *What Is a Dog?* How does this species we are interested in relate to other species that have been studied, or that need to be studied? What are the similarities and differences between them—and do they explain the similarities and differences between cognition as we see it in dogs, and cognition as we see it in other species? And, following on from that understanding, is there a unique contribution that the study of canine cognition can make to the study of comparative cognition in general?

We argue that we should look for comparison species for dogs in three different ways: phylogenetically, ecologically, and anthropogenically. That is to say, we need to ask what a dog is in terms of where it fits in to the great tree of descent from different and simpler organisms; what it is in terms of where it fits in to the complex web of resource-driven relationships that link all living things together in a system that is usually close to an equilibrium; and what it is in terms of its role in human history, and what is the human role in its history. We will, of course, be asking all those questions primarily about cognition. And we will be asking them about cognition as such, rather than about the neural mechanisms that subserve it, because we are not neuroscientists and do not wish to pretend to expertise we do not have. We take these three perspectives because, in our view, they represent the three great constraints on any species’ cognition. Phylogeny has a large influence on the kind of nervous system an animal possesses, the sensory inputs it can receive, and the kinds of motor responses it can make—the raw material on which cognition, and cognitive evolution, can work. Ecology specifies the purposes to which cognition is put in the natural life of an animal, and hence provides the potential motor for cognitive evolution. And, finally, in this Anthropocene age, humans modify every animal’s life chances, directly or indirectly—and in the case of domestic animals like dogs, we have molded them to our purposes in sometimes dramatic ways.

The structure of the paper is as follows. The first three sections position dogs on each of three dimensions: phylogenetically, as members of the order Carnivora; ecologically, as recently descended from cursorial social hunters; and anthropogenically, as domestic animals. We argue that these three perspectives between them define the dog, and suggest other species from which we should provide comparative studies, as illustrated by the Venn diagram shown in Fig. 1. The dog, in the middle, arguably occupies a unique position. The task of this paper is to explore whether that unique position makes the dog’s cognition exceptional, or whether its cognition is what we would expect from its membership of one or more of these three overlapping groups. We use the word “exceptional” in its ordinary sense of being far from the average or the predictable trend—usually far better. So we are asking whether dogs are more cognitively capable than could reasonably be predicted. Are they as special as many recent publications seem to imply?

**Fig. 1:**
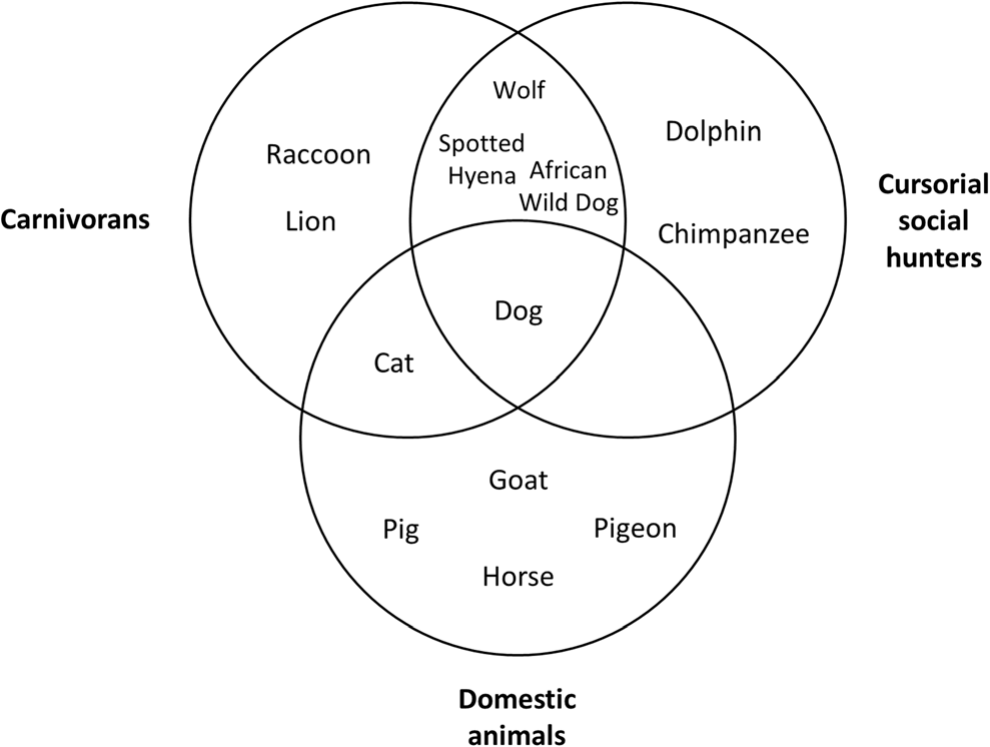
Phylogenetic, ecological, and anthropogenic groupings of species discussed in the paper

We pursue this task in the section of the paper titled "The Comparative Project", where we provide examples of studies of a variety of domains of cognition both in dogs and in comparison species, which occupy other regions of the diagram in Fig. 1. For each domain, we evaluate the position of dog cognition, as either similar or different to that of the comparator species. Subsequently, in a section entitled "The Comparative Intelligence of Dogs", we draw those evaluations together and seek to draw a conclusion about the special nature, or otherwise, of dog cognition. Finally, in a section entitled "The Contribution of Studies on Dogs to Our Knowledge of Comparative Cognition", we reflect on the contribution that recent studies of dog cognition have made to our understanding of comparative cognition in general.

## The phylogenetic context of dog cognition

From a phylogenetic perspective, dogs are members of the mammalian order Carnivora (see Wang & Tedford, 2010, for a detailed evolutionary history of the dog). We will refer to them as being carnivorans, because the obvious word “carnivore” is ambiguous; it can be used to mean a member of the order Carnivora, or to mean any animal that eats animal flesh. Although the order Carnivora gets its name from the fact that, unlike all other mammalian orders, most of its members eat animal flesh, there are a few carnivorans that are not carnivores in this more general sense (e.g., giant pandas), and many carnivorous animals that are not mammalian carnivorans, toothed whales and birds of prey being only the most obvious examples.

Wozencraft (2005) recognizes 286 species of carnivoran. The order is divided into two suborders: the cat-like feliforms and the dog-like caniforms. Each has several families within it: as well as the felids, the feliforms include civets, linsangs, hyenas, and mongooses, whereas the caniforms include bears, seals and sea lions, the red panda, skunks, mustelids and raccoons. But despite the number of other carnivoran species, the world population of dogs, estimated at 400 to 1,200 million (Coppinger & Coppinger, 2016) comfortably exceeds that of all other carnivorans combined. Only the domestic cat comes close, with a world population recently estimated at 600 million (Gehrt, Riley, & Cypher, 2010).

In seeking to place dog cognition into its phylogenetic context, therefore, we would like to see how far the cognition of dogs is similar to, or different from, that of the other 250–300 members of the order. But this ideal endeavor faces a snag. Just as the world population of dogs comfortably exceeds that of almost all other carnivorans combined, so too the world literature on dog cognition comfortably exceeds that on the cognition of all other carnivorans. In other words, our knowledge of carnivoran cognition derives very largely from studies on dogs. Current interest in understanding dog cognition has itself led to numerous studies of wolves, and in particular to comparisons of cognitive performance between dogs and wolves. It is not clear, however, that this comparison serves our present purposes well. At least within the biological species concept of Mayr (1942), there is no doubt that dogs are conspecific with wolves: Under Mayr’s concept, two populations are conspecific if the two populations hybridize freely and the hybrids are fertile, and this is true of dogs and wolves, though hybrids are rarely found in the wild (Vilà & Wayne, 1999). Admittedly, such hybridization is possible across a wide range of the genus *Canis*; however, genomic studies leave little room for doubt that dogs are descended from wolves and not from any other member of the genus (Ostrander, Wayne, Freedman, & Davis, 2017). Accordingly, in the present paper, we shall not be focusing strongly on the cognitive differences between dogs and wolves (which have in any case already been thoroughly explored in recent literature), but rather on the differences between dogs and wolves, considered together, and other carnivorans. This is a kind of comparison that has drawn much less attention.

So what other carnivoran species have been the subjects in studies of cognition? Arguably the earliest true experiment on animal problem solving used cats as subjects (Thorndike, 1898), and in numerical terms, studies of cat cognition probably do stand in second place to dog studies within carnivoran cognition. That is especially true if we take into account literature whose primary purpose was to investigate brain mechanisms; compared with those of dogs, cats’ skulls and brains are highly consistent in size and shape, so they are more convenient subjects for neurophysiological investigation. There is also a substantial literature on brain-damaged ferrets. However, it is often difficult to extract the true cognitive content from that kind of literature, so for the purposes of this paper we will set it aside. Beyond these domestic species, there is a significant recent literature on some aspects of the cognition of spotted hyenas, of some bears, and of some pinnipeds. There is also an older literature on raccoons (for which see Pettit, 2010).

The predominance of dog studies within the literature of carnivoran cognition does mean that there are some cognitive domains in which we know a fair amount about dogs, but very little about other carnivorans, so locating those aspects of dog cognition within carnivoran cognition will be difficult. However, as we shall see, it is possible to identify a number of cognitive domains in which we have significant evidence both about dogs and about one or more other species of carnivorans.

## The ecological context of dog cognition

The question of what a dog *is*, ecologically, is not a simple one. On the one hand, we can turn to its closest wild relative, the gray wolf, *Canis lupus*. On the other hand, the process of domestication has obviously changed the ecological niche of dogs in multiple ways. Many authors argue that the path toward full domestication involved a stage of scavenging around human settlements (e.g., Coppinger & Coppinger, 2001; Driscoll, Macdonald, & O’Brien, 2009). Few modern dogs get their living in the same way as wolves, or indeed as these primeval scavengers (Macdonald & Carr, 1995; Vanak & Gompper, 2009). The great majority of modern dogs, in fact, are “village dogs,” living in tolerated association with humans, partly provisioned deliberately by their human neighbors and partly scavenging (Coppinger & Coppinger, 2016, Chapter 2); and of those dogs that do hunt, they frequently do so individually. There is in fact scant evidence that any social hunting by free-living dogs is coordinated, or more successful than individual hunting (Boitani & Ciucci, 1995; Butler, Du Toit, & Bingham, 2004; Krauze-Gryz & Gryz, 2014). And then there is the minority of what we might call fully domesticated dogs, living as companion or working animals and with virtually all their needs provided intentionally by humans; and, bringing the domestication story full circle, some of these fully domesticated dogs work as social hunters. It is the minority group of companion and working dogs that provides the subjects for most studies of canine cognition; but which of these four ecological niches truly defines a dog?

Consistently with the approach we have taken for phylogenetic comparisons, in this paper we will focus our ecological comparisons primarily on the niche occupied by wolves. Of the other three possibilities, the primeval pure scavenging role is essentially speculative, and it would be difficult to define it precisely. The “village dog” and companion/working animal roles deserve fuller consideration. Their leading characteristics are omnivory, the need for tolerance of human presence, and partial or total provisioning by humans. These changes may well have cognitive implications, and we will bring them into our discussion through our third perspective on the nature of dogs: the anthropogenic context. However, dogs retain the predatory action sequences seen in wolves (Coppinger & Coppinger, 2001, Chapter 4); it is not unreasonable to suppose that they also retain the cognitive mechanisms required for social hunting. Indeed, the fact that some working breeds are selected as social hunters supports this supposition.

In terms of its foraging ecology, the gray wolf is an unusual caniform, and even an unusual member of its genus: although it is, like most carnivorans, carnivorous, and like most canids, a hunter rather than a sit-and-wait predator, it is unusual in being gregarious and hunting in packs. Put briefly, wolves are social cursorial hunters. As a result, they are able to take prey substantially larger than themselves, which is atypical though not unique among the Carnivora. As a further consequence, they live in relatively large, relatively organized packs (Mech & Boitani, 2003), which is again atypical though not unique among the Carnivora.

What other animals share this way of making a living, and hence of living together? Social hunting within the Carnivora has been valuably reviewed by Bailey, Myatt, and Wilson (2013), who consider a wide range of possible examples; here, we will focus only on the most salient. Certainly some of the other closely related canids, such as the red wolf and coyote, do also hunt socially. But they do so only occasionally, and there is little to suggest that they often, if ever, engage in the lengthy pursuits of prey that characterize wolves’ hunting (Bekoff, 1977; McVey et al., 2013). The closest in behavior to the gray wolf are the dhole of Asia and the African wild dog, both of which routinely hunt in packs and take prey larger than themselves (Hayward, Lyngdoh, & Habib, 2014; Hayward, O’Brien, Hofmeyr, & Kerley, 2006). Among the large feliforms, the closest in behavior to the wolf is the spotted hyena, which hunts large prey socially (Hayward, 2006); among the felids, only lions hunt socially, and they are not cursorial predators in the same way as wolves, using ambush tactics instead (Stander, 1992). Cheetahs also sometimes hunt together, and when they do, they can take large prey, but it is only coalitions of two that are seen, rather than packs, as in wolves and hyenas (Broekhuis, Thuo, & Hayward, 2018). Some solitary feliforms do take prey substantially larger than themselves: leopards, for example, have been known to kill eland, typically several times their weight (Bailey, 2005), but they are hide-and-pounce rather than cursorial predators. Most other feliforms are solitary hunters, and in consequence tend to take prey substantially smaller than themselves.

What about noncarnivoran carnivores? The animal from another order most closely resembling a wolf was the thylacine, or marsupial wolf, but that unfortunately became extinct before its behavior could be properly studied; it may possibly have hunted in small social groups, but biomechanical examination leads to the conclusion that, like extant carnivorous marsupials, it mainly ate prey much smaller than itself (Attard, Chamoli, Ferrara, Rogers, & Wroe, 2011; Figueirido & Janis, 2011). Among other mammalian orders, the obvious examples of social cursorial hunters are some of the toothed whales, with the bottlenose dolphins having been well studied; they use organized group hunts to attack large shoals of fish (e.g., Gazda, Connor, Edgar, & Cox, 2005). Another significant case for our purposes are the chimpanzees. Although meat forms only a small portion of their diet, both common chimpanzees and bonobos have been observed taking part in group hunts, with prey that are at least close in size to themselves, such as colobus monkeys and mangabeys (e.g., Stanford, Wallis, Matama, & Goodall, 1994; Teleki, 1973, for common chimpanzees; Surbeck & Hohmann, 2008, for bonobos).

As regards the other vertebrate classes, group hunting of any sort is rare in birds, though it has been documented for Harris hawks (Bednarz, 1988) and brown-necked ravens (Yosef & Yosef, 2010). Hector (1986) summarizes literature showing that hunting in groups is not uncommon among raptors, but mostly it is not truly social in that there is no sign of cooperation, such as labor division, signaling, or sharing of food. Success rates are no higher, and prey sizes no greater, than in solitary hunting. However, Hector collects a number of reports of true cooperative hunting, though mainly between pairs rather than larger groups; his own study of the Aplomado falcon is an example. Among cold-blooded vertebrates, there are reports of cooperative group hunting in crocodilians (Dinets, 2015), boas (Dinets, 2017), and at least one teleost, the zebra lionfish (Rizzari & Lönnstedt, 2014). All of these hunts showed clear signs of cooperation, involving specialized roles, turn taking, and occurred more than once involving the same grouping of individuals. Many species of shark also hunt in groups, but in these cases there seems to be competition rather than cooperation between the group members (e.g., Hobson, 1963; Robbins & Renaud, 2016). We have not been able to trace any literature on cooperative pursuit in invertebrates, though it would be surprising if there were none.

In summary, although we have not found as many other cursorial social hunters as we found other carnivorans, from the point of view of a comparative approach to dog cognition, the ecological comparison set looks rather more promising than the phylogenetic one. It includes at least two very well-studied species, the chimpanzee and the bottlenose dolphin, and one, the spotted hyena, for which we have quite substantial information, albeit mostly from a single extended research program (Holekamp, Sakai, & Lundrigan, 2007).

We have focused here on wolves' and dogs’ foraging ecology, rather than their social structure or reproductive systems, which are also important aspects of any species’ ecology, and which can undoubtedly play a part in the evolution of cognitive capacities. We agree with the position of Wrangham (1979) that foraging ecology is fundamental to all social relations, but certainly some aspects of cognition are more easily, and more proximally, predicted from other observable aspects of society. For example, the need to keep track of individuals and their relative dominance in a large society has been considered a crucial influence in the evolution of larger and more capable brains (see, for example, Dunbar & Schultz, 2007; Humphrey, 1976). Similarly, the need for one sex to navigate in a large territory within which several potential mates may be found, as in some forms of polygyny or polyandry, has been considered to explain sex differences in spatial cognition (Gaulin & Fitzgerald, 1986). For our purposes, however, it is most appropriate to focus on comparisons with social hunters, rather than (for example) all animals who live in groups with substantial variations in dominance. In the first place, such a comparison group would be impracticably large; more to the point, however, it is precisely in relation to hunting that it has been argued that wolf and dog cognition may have been affected by social pressures, through the demands for cooperation that social hunting creates (e.g., Range & Virányi, 2014). The idea that hunting might lead to enhanced cognition has a long history, not least in relation to human evolution (e.g., Washburn & Lancaster, 1968). Yet the strategies that determine social hunting in wolves can be simulated using two very simple rules (Muro, Escobedo, Spector, & Coppinger, 2011), and therefore the relationship between social hunting and enhanced cognition might not be as strong as proposed. It would be interesting to compare wolf and dog cognition with that of species that have similar societies to wolves or dogs, but very different foraging ecology; that, however, is beyond the scope of the present paper.

We do need to reiterate, however, that we have been looking at ecological comparators for the behavior of wolves, and, as we saw above, this is only one out of four possible ways of viewing the dog’s ecological niche. The process of domestication has involved significant ecological shift, and it is widely thought to have been preceded by a shift toward a scavenging way of life. Accordingly, we now turn to consider what species we should compare with dogs if we consider them as domestic animals.

## The anthropogenic context of dog cognition

Our final way of defining a dog is anthropogenically. The dog is an animal that has been domesticated by humans, so it makes sense to compare it with other domesticated animals. Of course, different animals are domesticated for different purposes, and we might well ask what we could expect dogs to have in common, cognitively or in any other way, with species like pigs that are kept for meat; horses that are kept for physical work; cows, sheep, and chickens that are kept to allow their bodily products to be harvested; and cats and cage birds that are kept for aesthetic reasons or companionship. However, all these species are actually kept for more than the purposes we have indicated, and dogs are or have been kept for all of them, to greater or lesser extents. And certainly the purposes for which most dogs are kept nowadays are different from those for which they were first domesticated—which from current archaeological evidence was later, but not much later, than 16,000 B.P. (Perri, 2016).

Despite these differences, domestic animals tend to share certain differences from their wild ancestors (Price, 1999)—what Wilkins, Wrangham, and Fitch (2014) call the “domestication syndrome.” Many of these features are morphological (e.g., reduced size, shorter muzzles, smaller teeth), but some are behavioral. Increased tolerance for the close presence of humans, and, indeed, of other animals, both conspecifics and others, is an important example. Individuals of entirely wild species can acquire such tameness, especially if they live with humans from birth, but there is at least some evidence that species that have been long bred in captivity acquire it more quickly and fully than wild conspecifics, a point that will become relevant when we turn to the cognitive comparison of dogs and wolves. Reduced dependence on active foraging for food is another common characteristic of domestic animals, and with it almost certainly increased tolerance of variations from the ancestral diet and of unusual foods or ways of acquiring food—the last a fact that can be useful in devising cognitive experiments. And finally, we should remember that virtually all domestic animals have been subject to deliberate artificial selection (as well as natural selection for the traits we have just listed), for all sorts of traits, some of which may have consequences for their cognition or at least for the performance of the tasks by which we seek to evaluate cognition.

The domestic animals afford a number of interesting cases for cognitive comparison with dogs. In addition to cats, which we have already noted for phylogenetic reasons, there are good reasons for looking in particular at horses, because, like dogs, they are commonly submitted to elaborate training; and pigeons, because they have been subjects in a huge number of cognitive experiments. Birds of prey used in falconry are an anomalous case: They are interesting because, like dogs, they are used in cooperative hunting with humans, but they are tamed rather than domesticated: Until very recently, all birds used in falconry were captured from the wild as chicks (Gallagher, 2008). Accordingly, we cannot consider them as an appropriate comparison group. Domestic elephants also have normally been captured from the wild, so we have not included them, either. Pigeons might not seem an obvious case, perhaps because the widespread presence of feral pigeons leads us to forget that pigeons have a longer history of domestication than any other bird except the chicken, both having been domesticated something between 5,000 and 10,000 years ago (R. F. Johnston, 1992; West & Zhou, 1988)—although Neanderthal humans were eating the pigeon’s wild ancestors in substantial numbers thousands of years earlier (Blasco et al., 2014). In addition, a useful experimental literature is now collecting on the cognition of other domestic species, including sheep, pigs, and goats.

## The comparative project

In order to set dog cognition into its comparative context, therefore, we will focus on comparing dogs with the following species, on all of which at least some cognitive literature is available:

- Wolves, as the wild ancestor, and closely related members of the genus *Canis* (though, as explained above, for many purposes we shall consider dogs and wolves together)
- African wild dogs and spotted hyenas, as both carnivorans and social hunters
- Cats, both as carnivorans and as domestic animals
- Bottlenose dolphins and chimpanzees, as social hunters
- Horses and pigeons, as domestic animals

While we believe the list gives us good coverage without making for an impossibly extensive presentation, we also include studies on a miscellany of other carnivorans, social hunters, and domestic animals, in cases where relevant literature is available on them and not on our target comparison species.

It is worth noting some taxa that do not appear on this list. We will not be attempting to survey the cognition of primates in general, corvids, parrots, rats, bats, cleaner fish, bees, or jumping spiders—not because the cognition of those species is not interesting, but because within our present framework there is no immediate reason to compare it with the cognition of dogs. However, there is one other species that we must bear in mind: humans. While humans are not carnivorans, we match dogs in that ancestrally, and indeed for the majority of our existence as a species, we were social hunters. Furthermore, anthropologists argue that humans have been the subject of a domestication process that parallels that undergone by the species we own and use (e.g., Leach, 2003). Most important, however, we are the reason why the dog has come to differ from the wolf. Furthermore a number of the tasks used in testing cognitive abilities in dogs were first devised for humans—often young humans. One of the basic reasons for being interested in animal cognition is to understand more precisely what is and is not unique about human cognition; and as part of that project, there is a lot more point in comparing human cognition with the cognition of dogs than with the cognition of, say, pigeons—even though that, too, may sometimes be worthwhile (e.g., Maes et al., 2015; Wills et al., 2009).

In the main body of this paper, therefore, we compare the results available from dogs with those from the other taxa we have identified, across a range of domains of cognition and cognitive tasks within them; and in our concluding section we will weigh up the evidence we have gathered, to draw an interim conclusion about how special dog cognition appears to be. It can only be an interim conclusion, because in the present state of our knowledge, our comparison will inevitably be incomplete. Furthermore, its coverage is inevitably uneven. Whereas for some of the comparison species, we could usefully sweep in everything that is known about their cognition, for others—especially chimpanzees and pigeons—we can only pick out the most salient points from a vast literature. Nor are all domains of cognition considered here: In particular, to keep things tractable, we have left to one side studies of abstract reasoning, number, and time sensitivity; and we have not considered memory as a separate category, although it does, of course, enter into studies of many of the cognitive phenomena we are considering.

In dividing our material into a number of different cognitive domains, we are not implying that these correspond to distinct cognitive mechanisms, let alone cognitive “modules” in the sense in which some evolutionary psychologists have used that concept (e.g., Fodor, 1983), that is, cognitive capacities that are independently evolved and restricted to a particular task. If particular species show distinctive cognitive capacities, that might be taken as implying that their distinctive ecological niche has led to the evolution of a distinct cognitive module to subserve them, as Fodor assumed was the case with human language. But, although we do expect to find some cognitive differences between species, we make no assumptions about the existence of strict domain specificity, and in our view the data are not yet available to test such a view.

### Associative learning

Many experiments on animal cognition have to start by establishing some behavior through simple associative learning—habituation, Pavlovian conditioning, operant conditioning, or avoidance learning. That behavior is then used in assays of more complex, or supposedly more advanced, cognitive processes. If there were differences between dogs and other species in their response to these basic procedures, we would need to understand them before we could consider anything more complex. So we need to consider them first. However, in fact we need say very little about them. The basic forms of associative learning have all been investigated thoroughly in dogs—operant conditioning less thoroughly than the others in the laboratory, but extensively in the applied context of training working and show dogs. No other carnivoran has been investigated as thoroughly in any of them, but from the limited evidence to hand (e.g., on avoidance conditioning in cats; e.g., McAdam, 1964; Seward & Humphrey, 1967) we can reasonably conclude that, so far as we yet know, both dogs and other carnivorans show these simple forms of learning in the same way as other vertebrate species. The same is true of the other social hunters we identified, and of other domestic species: indeed, the pigeon is as stereotypically identified with operant conditioning as the dog is with classical conditioning.

Although the basic processes of associative learning can be observed in all vertebrate species, that is not to say that the details are the same for all species and all situations. It is well accepted that the so called “laws of learning”, applying especially to what is usually called conditioning, show species-specific variations at least in their parameters (Hinde & Stevenson-Hinde, 1973). These are often referred to as variations in “preparedness,” meaning that there is an evolved link between stimulus, response, and reinforcer, which makes their association easy to learn (Seligman, 1970). Seligman listed examples of variations in preparedness in almost all the species widely used in the study of learning; and dogs are no exception. For example, Jenkins, Barrera, Ireland, and Woodside (1978) showed that dogs had a “prepared” association between food and licking. But although prepared associations take different forms in different species (Domjan & Galef, 1983), the fact that variations in preparedness are so widespread means that it would be more remarkable if there were no examples in dogs. In an early paper, Frank (1980) claimed that there were differences in associative learning between dogs and wolves, with wolves being more susceptible to operant conditioning than dogs, but this generalization has not stood the test of time and replication, and the differences observed may well have been due to differences of rearing conditions (see Frank, 2011). In summary, without even making use of our comparison groups, we can conclude that there is no evidence that associative learning is in any way unusual in dogs.

### Sensory cognition

The other foundational way of looking at animal cognition is to start with the stimulus input, and consider what sensory resources an animal has (perception), and what information it can extract from the perceptual input (sensory cognition). We will consider the major senses in turn.

#### Olfaction

Comparative psychophysical data on thresholds for olfaction between dogs and other species of interest do not exist (Wackermannová, Pinc, & Jebavý, 2016). In any case, it is more important for our purposes to examine what dogs, and other animals, can do with their olfactory input. The discriminative olfactory capacities of dogs are remarkable, extending to discriminating the direction in which a scent trail has been laid (Wells & Hepper, 2003), and between any two human individuals, even, under at least some conditions, monozygotic twins (Hepper, 1988; Kalmus, 1955; Pinc, Bartoš, Reslová, & Kotrba, 2011). Dogs can also be trained to assign dissimilar odors to a single category (Wright et al., 2017). They also seem to form a representation of what they smell, as Bräuer and Belger (2018) demonstrated using a violation-of-expectation experiment. Despite their outstanding olfactory discrimination, however, dogs are not necessarily dominated by olfactory information: Human pointing can override olfactory cues in some situations (Szetei, Miklósi, Topál, & Csányi, 2003).

Unfortunately, we have not been able to find anything like comparable tests of olfactory ability with any other carnivoran or social hunter. Cats can discriminate their own from others’ kittens by smell (Banszegi, Jacinto, Urrutia, Szenczi, & Hudson, 2017), and it would be surprising if this was not true of many other carnivorans. Although behavioral data are lacking, we do have comparative anatomical data, showing that large canids, including the wolf, have disproportionately large olfactory turbinal surface areas (the nasal structures that allow olfaction) compared with most other carnivorans, except for other large, high-latitude carnivores such as the polar bear (Green et al., 2012); conversely, marine carnivorans (i.e., pinnipeds) have much lower olfactory turbinal surface areas (Van Valkenburgh et al., 2011). Among domesticated animals, the pig’s olfactory abilities are outstanding and might even be better than the dog’s (Nguyen et al., 2012), and pigs can also discriminate between familiar and unfamiliar people’s smell (Tanida & Nagano, 1998). And horses can identify conspecifics based on feces smells (Krueger & Flauger, 2011). So the olfactory performance of dogs is not that extraordinary among two of their comparison groups, carnivorans and domestic animals.

#### Gustation

In taste, dogs outperform cats, in that cats, and, indeed, all felids tested seem to be entirely insensitive to sweet taste for genetic reasons, while dogs have a different genetic structure in the relevant area and do respond to sweetness (Li et al., 2009; Li et al., 2006); since the ultimate reason for cats’ neglect of sweetness is claimed to be their obligate carnivory, it is likely that dogs also outperform many other carnivorans of which the same would be true, though probably not the omnivorous carnivorans such as badgers and bears.

#### Mechanoreception

Dogs may be less sensitive than some other carnivorans in the whisker sense, which seems to be particularly important in aquatic carnivorans and has been well studied in seals (see Hyvarinen, Palviainen, Strandberg, & Holopainen, 2009). For cats the vibrissae are an important tactile sense; their removal results in impaired locomotion (Schmidberger, 1932). There is neurological evidence that whiskers fulfil important tasks in dogs (McGill, 1980), but we have found no behavioral data to confirm this.

#### Vision

The field of basic visual perception is too vast to attempt to summarize here, but we are not aware of anything to suggest that dogs are exceptional among carnivorans, either positively or negatively, at the basic perceptual or psychophysical level. Selective breeding by humans has affected the layout of dogs’ retinas and the neural connections within them, but the changes seem to be in the direction of conserving function rather than modifying it. In any case, as with the other senses, our interest is primarily in what animals can do, cognitively speaking, with their visual input. Although the field of visual cognition is not particularly well developed for any carnivoran, we do know that dogs can discriminate complex visual patterns, showing abilities comparable to those that have been shown more fully for pigeons, primate species, and some domestic ungulates. For example, a commonly used discrimination of this kind is between the faces of individual humans, or between facial expressions, and this is something that dogs can do (for examples, see Racca et al., 2010, and Somppi et al., 2016). Similar abilities have been found in chimpanzees (e.g., Martin-Malivel, & Okada, 2007; Parr, Winslow, Hopkins, & De Waal, 2000), pigeons (e.g., Troje, Huber, Loidolt, Aust, & Fieder, 1999), and sheep (e.g., Kendrick et al., 1995; Kendrick, da Costa, Leigh, Hinton, & Peirce, 2001). There is less evidence for other carnivorans, but it is positive: For example, Fields (1936) gave a simple demonstration of visual object discrimination in cats, and black bears have been trained to make both perceptual and more abstract category discriminations (Vonk, Jett, & Mosteller, 2012; Vonk & Johnson-Ulrich, 2014). Vonk and Leete (2017) have recently reviewed evidence for categorization across the carnivorans and have concluded that the capacity is fairly widespread in the order.

There are some tests of visual cognition in which pigeons have been found to behave differently from humans, suggesting that they see the test stimuli differently. In these tests, dogs have been found to behave more like humans. For example, if one object is partly hidden (occluded) by another, pigeons do not respond to it as though the entire object was still there (Sekuler, Lee, & Shettleworth, 1996); but dogs, like humans, apparently recognize the continuing solidity of the occluded object (Pattison, Miller, Rayburn-Reeves, & Zentall, 2010). Similarly, pigeons tend to respond to hierarchical stimuli (in which a whole is made up of differently shaped elements) in terms of their elements (Cavoto & Cook, 2001), whereas dogs, again like humans, respond to face stimuli more in terms of their configurational properties than their constituent parts (Pitteri, Mongillo, Carnier, & Marinelli, 2014). Among our comparison species, cats have been shown to perceive subjective contour, a task somewhat similar to dealing with occluded stimuli (Bravo, Blake, & Morrison, 1988).

Among other social hunters, dolphins tend to behave like humans in an echolocation analogue of the hierarchical stimulus test (Pack, Herman, Hoffmann-Kuhnt, & Branstetter, 2002), while chimpanzees, like dogs, behave more like humans on the occluded stimulus task than pigeons do (Sato, Kanazawa, & Fujita, 1997). However, the evidence on chimpanzee performance with hierarchical stimuli is inconsistent (contrast Hopkins & Washburn, 2002, with Fagot & Tomonaga, 1999).

More elaborate tests of visual cognition include the categorical same/different discrimination, that is, the ability to respond discriminatively to pairs of stimuli according to whether they are the same or different, regardless of what the stimuli are, and in particular to transfer that discrimination to stimuli that have never been seen before. The only claim of such an ability in dogs that we know of used rather few stimuli, and it was found only in the auditory, not in the visual domain (Pietrzykowska & Soltysik, 1975a, 1975b). In the auditory domain, dogs were able to make differential responses to sequences of the same stimulus (using a variety of different sounds, such as white noise, clicks, and metronome beats) rather than sequences in which the sound varied, but in the visual domain, using continuous versus rhythmically pulsing lights, they failed to discriminate. Categorical same/different discrimination has been demonstrated more convincingly in pinnipeds, both in a common seal (Mauck & Dehnhardt, 2005) and in a sea lion (Hille, Dehnhardt, & Mauck, 2006); the seal even transferred the concept from shape discrimination to pattern and brightness discriminations (Scholtyssek, Kelber, Hanke, & Dehnhardt, 2013). Categorical same/different discrimination seems to be spontaneous in chimpanzees (Oden, Premack, & Thompson, 1988), but is only acquired after extensive training with pigeons (Wright, Cook, Rivera, Sands, & Delius, 1988).

#### Audition

Basic auditory perception is also too large a field to embark on here, not least because we are not aware of any evidence that dogs’ auditory abilities, though evidently good, are exceptional. In terms of auditory cognition, it is obvious from the large number of object names that one or two dogs have been trained to discriminate (Kaminski, Call, & Fischer, 2004; Pilley & Reid, 2011) that dogs can discriminate human speech sounds; and other experimental work confirms this (Baru & Shmigidina, 1976; Ratcliffe & Reby, 2014). Evidence for discrimination of human speech sounds in the everyday life of other carnivorans is less obvious, but there are experimental demonstrations of discrimination of aspects of human language, for both cats (Hienz, Aleszczyk, & May, 1996—vowel discrimination) and ferrets (Bizley, Walker, King, & Schnupp, 2013—timbre discrimination). In chimpanzees, the various experiments on human language learning have demonstrated robust discrimination of substantial numbers of spoken words, on the order of 100 for both a chimpanzee and a bonobo (Brakke & Savage-Rumbaugh, 1995), even at a relatively early stage of the project. Horses and some other domestic animals must be able to discriminate at least a few human speech sounds, used as commands, but we have not found any quantitative estimates of their vocabulary.

Both dogs and cats have been shown to discriminate between the voices of different humans (e.g., Coutellier, 2006; Saito & Shinozuka, 2013), but we have not found any quantitative estimates of the number of humans who can be recognized, or the robustness of the discriminations. Recognition of individual conspecifics by voice appears to have been relatively neglected in dogs, though it has been demonstrated in some other carnivorans, including the spotted hyena (Holekamp et al., 1999), the domestic kitten (Szenczi, Bánszegi, Urrutia, Faragó, & Hudson, 2016), the Asian short-clawed otter (Lemasson, Mikus, Blois-Heulin, & Lode, 2013), the dwarf mongoose (Sharpe, Hill, & Cherry, 2013), and pinnipeds (e.g., Pitcher, Harcourt, & Charrier, 2010; Van Parijs & Clark, 2006). It is highly developed in dolphins, through the use of “signature whistles” (Tyack, 1997), and also in some domestic animals: For example, in sheep it is used very early in life (Sèbe, Duboscq, Aubin, Ligout, & Poindron, 2010), and horses have been shown to link the voices of individual conspecifics with their visual appearance, demonstrating a cross-modal concept of the other individuals’ identities (Proops, McComb, & Reby, 2009).

#### Summary

It appears, therefore, that the perceptual abilities of dogs do not differ from what we would expect from our comparison groups. Their olfactory abilities are excellent, but similar abilities are found in some other carnivorans and domestic animals. Their sensory cognition seems to be similar to that of other carnivorans and social hunters that have been tested, and to that of some but not all domestic animals.

### Physical cognition

By physical cognition, we refer to an animal’s capacity to operate effectively on the world of objects—generally, objects smaller than, or comparable in size to, themselves. Research in this area has been strongly influenced by ideas and procedures first devised for the investigation of human cognitive development, and especially for testing the theory that cognitive development proceeds in discrete stages, as put forward by Piaget and his collaborators (see Doré & Dumas, 1987, for further details of the incorporation of these ideas into comparative cognition). This approach has given us a number of more or less standard tasks that have been successfully adapted for use with a wide range of species.

The simplest question that has been posed to animals within the Piagetian framework is that of object permanence—that is, whether the animal appears to know that an object that has disappeared from view (or from the range of other senses) continues to exist. Early experiments on object permanence on dogs, using tasks in which objects were displaced either visibly or invisibly (within a container), led to claims that adult dogs performed up to the highest level (Stage 6) of Piaget’s sensorimotor phase of development (Gagnon & Doré, 1992; Triana & Pasnak, 1981), and that performance at this level was shown from 8 weeks of age (Gagnon & Doré, 1994). In a recent review, Zentall and Pattison (2016) have reaffirmed this position. Most authors, however, now take a more nuanced stand. The original experiments, and subsequent work (e.g., Fiset, Beaulieu, & Landry, 2003) leave no doubt that dogs continue to search for an object that has disappeared. However, in general, dogs have been found to do less well in tasks involving invisible displacement of a hidden object, and it is success in these tasks that underpins the claim for Stage 6 performance. Dogs’ behavior in such tasks does not correspond to that of human children (Watson et al., 2001) or great apes (Rooijakkers, Kaminski, & Call, 2009), and it can be strongly affected by details of the procedure, especially the social interactions (Topál, Gergely, Erdőhegyi, Csibra, & Miklósi, 2009).

Most recent experimenters (Collier-Baker, Davis, & Suddendorf, 2004; Fiset & LeBlanc, 2007) concur that the basis for solution of invisible displacement tasks lies in associative learning rather than in a mental representation of the vanished object. However, Miller, Gipson, Vaughan, Rayburn-Reeves, and Zentall (2009) and Miller, Rayburn-Reeves, and Zentall (2009b) have argued, using an unconventional rotational task, that they do have evidence for object permanence during invisible displacement in at least some dogs. In their experiments, a beam with a box at each end was rotated around a central point, and the dogs were found to search in the box into which they had seen the desired object being placed; the performance of some dogs was unaffected by a delay before they were allowed to search, and Miller et al. therefore argued that these dogs must indeed have formed a mental representation. It is also possible that dogs’ poorer performance in other invisible displacement tasks was due to the displacement device used: Müller, Riemer, Range, and Huber (2014b) demonstrated that even in visible displacements, using such a device affected performance negatively. Even without invisible displacement, there are some puzzling results in experiments on dogs’ object permanence: For example, Müller, Mayer, Dorrenberg, Huber, and Range (2011) found that male (though not female) dogs seemed to be unsurprised if an object changed size while it was hidden, though in similar tasks neither Bräuer and Call (2011) nor Pattison, Laude, and Zentall (2013) reported any such sex difference.

How do our comparison groups fare in tests of object permanence? In a recent review of the literature, Jaakkola (2014) concludes that only great apes have shown evidence of understanding invisible displacement in object permanence tasks, though many species cope well with visible displacements. Wolves perform comparably to dogs (Fiset & Plourde, 2013); sea lions are successful in visible displacements (Singer & Henderson, 2015), and so are sloth bears (Amici, Cacchione, & Bueno-Guerra, 2017). The only other carnivorans that have been tested extensively are cats, and although they have been claimed to reach sensorimotor Stage 6 (Gruber, Girgus & Banuazizi, 1971; Heishman, Conant, & Pasnak, 1995), in direct comparisons they generally perform less well than dogs, especially on tasks involving invisible displacement (Goulet, Doré, & Rousseau, 1994). Among the other social hunters we have considered, only chimpanzees have consistently shown evidence of object permanence in both visible and invisible displacement tasks. Dolphins, like dogs, generally perform well in visible but not invisible displacement tasks (Jaakkola, Guarino, Rodriguez, Erb, & Trone, 2010; Singer & Henderson, 2015), though it has been claimed that with a more ecologically valid procedure they will succeed even with invisible displacement (C. M. Johnson, Sullivan, Buck, Trexel, & Scarpuzzi, 2015). Among domestic animals, object permanence in visible displacement tasks has been reported in both goats (Nawroth, Von Borell, & Langbein, 2015) and pigs (Nawroth, Ebersbach, & Von Borell, 2013).

Beyond object cognition, standardized tasks inspired by Piagetian developmental psychology form the majority of what are often called “animal problem-solving” situations. In such situations, the question being asked is usually not whether an animal can learn, or be trained, to solve a particular problem, but whether it does so spontaneously, on first encountering the problem—that is to say, whether its ordinary cognitive capacities, as refined by everyday experience under normal rearing conditions, give it the ability to “see” a solution immediately. This description reminds us of the other important root of studies into animal physical cognition, Köhler’s (1925) extended study of “insight” in chimpanzees (which in fact included a few experiments on other species, including dogs).

The general consensus of the literature is that dogs show little or no insight in physical cognition problems. Whether it is recognizing that a connecting tube will guide a falling object to a particular place (Osthaus, Slater, & Lea, 2003), pulling a string to obtain a treat attached to the end of it (e.g., Fischel, 1933; Shepherd, 1915; Osthaus, Lea, & Slater, 2005; Range, Moslinger, & Virányi, 2012), support tasks (Müller, Riemer, Virányi, Huber & Range, 2014), using the “solidity principle” to predict where a moving object will come to rest (Müller, Riemer, Range & Huber, 2014a), or opening a latch to escape from a box (Protopopov; see Windholz, 1999), either all dogs have been found to fail to solve the problem spontaneously, or only a minority have succeeded. Dogs can, of course, be trained to perform many of these tasks, but they do not solve them on the first trial as children do once they have passed a certain age. A particular case of physical cognition is the use of tools, and we have not found a convincing case of spontaneous tool use in a dog; the nearest is a claim by Smith, Appleby, and Litchfield (2012) that a captive dingo spontaneously moved a table around its enclosure in order to obtain out-of-reach food, echoing one of the tasks Köhler used with his chimpanzees. Once again, dogs can be trained to use tools, though scientific studies of such training are lacking.

Although this is a broad consensus, there are exceptions, and also dissenting voices. As already stated, dogs can learn to perform some of these tasks, even if they do not solve the problem immediately, for example, the support problem using planks (Müller et al., 2014). Dogs do solve the simplest string-pulling tasks, with strings leading directly to the target object; where they fail is with multiple strings placed obliquely, or crossing each other (Osthaus et al., 2005); and a string-pulling experiment with vertically hanging ropes gave more positive results than the usual horizontal string situation (Hiestand, 2011)—though wolves in the same situation did better than dogs. Dogs have done relatively well at spontaneously solving simple container-opening problems (Duranton, Rodel, Bedossa, & Belkhir, 2015). Tasks involving the location and nature of hidden objects are generally solved well, though not necessarily with any understanding of a hidden object’s trajectory (e.g., Collier-Baker et al., 2004).

Performance in physical cognition tasks is not necessarily uniform across all dogs and contexts. Wolves (Frank & Frank, 1985), highly trained dogs (Marshall-Pescini, Valsecchi, Petak, Accorsi, & Prato-Previde, 2008), clicker-trained dogs (Osthaus, Lea, & Slater, 2003), and dogs with a high level of inhibitory control (Müller, Riemer, Virányi, Huber & Range, 2016) are sometimes found to outperform ordinary pet dogs in problem-solving tasks, while dogs raised in a restricted environment do worse (e.g., Clarke, Heron, Fetherstonhaugh, Forgays, & Hebb, 1951). This variability in performance emphasizes the general point about the importance of having similar rearing conditions when making species comparisons.

As regards our comparison groups, there is evidence that at least some other carnivorans do better than dogs at some physical cognition tasks. Using experimentally naïve animals (as was the case with the dog) and similar apparatus, cats are no better than dogs at string-pulling problems (Whitt, Douglas, Osthaus, & Hocking, 2009), but raccoons seem to solve them easily (Michels, Johnson, & Pustek, 1961). Jacobs and Osvath (2015) provide a detailed summary of string-pulling demonstrations and experiments through the ages and across more than 160 species. Spotted hyenas appear to be skilled at physical problems (e.g., Benson-Amram, & Holekamp, 2012), as are meerkats (Thornton & Samson, 2012); however, Thornton and Samson are skeptical about the contribution of cognitive ability, rather than sheer persistence, to the solution of the problems they set their subjects. In their comprehensive review of tool use in animals, Bentley-Condit and Smith (2010) list a few reports of carnivorans showing what they consider to be true tool use, including giant pandas, a lion, American badgers, and two species of bear, in addition to the well-known case of sea otters using stones to open clam shells (Hall & Schaller, 1964). A further experimental report on tool use in brown bears has appeared since that review (Waroff, Fanucchi, Robbins & Nelson, 2017); and Lindsey, du Toit, and Mills (2004) report that African wild dogs (a species on which we have almost no cognitive data) learn to use fences to help them trap larger prey than they could otherwise catch. The most extensive report on physical cognition in carnivorans comes from Benson-Amram, Dantzer, Stricker, Swanson, and Holekamp (2016). They tested a total of 140 individuals from 39 different carnivoran species on standard puzzle boxes, varying only in size. The numbers of individuals in each species tested were small, so Benson-Amram et al. focus their analysis at the level of species and families, and demonstrate that species with larger brains relative to body mass tended to have greater success. However, the authors provide their complete data set, which we have used to address more detailed questions of interest for this paper. According to these data, different individuals were given different numbers of trials, but almost all were given at least three trials, and the variations in performance within this range are striking. None of the three wolves tested solved the task on any of the first three trials. Almost all other canids also failed on all three: the only exceptions were two Arctic foxes (out of five tested) and two (out of five) African wild dogs. Representatives of some other families were substantially more successful, with six out of eight North American river otters, six out of seven coatis, four out of five black bears, three out of three brown bears, and three out of four snow leopards solving the problem at least once in their first three trials. Unfortunately, Benson-Amram et al. did not include any domestic dogs in their sample, so we do not know whether dogs would have done as badly as the wolves. Borrego and Gaines (2016) conducted another substantial cross-species puzzle box study, which demonstrated that several large feliforms successfully solved the problem within three trials; of the species they tested, spotted hyenas were the most successful, and tigers the least, with lions and leopards intermediate. Once again, however, we have no directly comparable data from dogs.

The social hunters also include some species with apparently more advanced physical cognition than dogs. As well as hyenas, mentioned above, raptors have also been found to solve some physical problems, for example, string pulling (a Harris hawk: Colbert-White, McCord, Sharpe & Fragaszy, 2013) and box-opening (chimango caracaras: Biondi, Bo & Vassallo, 2008). And despite their inability to manipulate anything with their limbs, bottlenose dolphins have been found to use tools (Krützen, Mann, Heithaus, Connor, Bejder, & Sherwin, 2005). Chimpanzees are famously manipulative, though Köhler (1925, pp. 27–30) found that the one chimpanzee he tested did not do well at complex string-pulling tasks, falling into the same kind of proximity errors as have been observed in dogs. However, chimpanzees’ spontaneous tool use in the wild has been thoroughly documented and studied since it was first reported by Goodall (1964), so there is little doubt of their capacity for at least some kinds of physical cognition.

Physical cognition has not been extensively studied in domestic animals other than cats, at least formally, and most reports of problem solving by farm animals demonstrate little if anything more than basic operant conditioning. In a comprehensive review of cognition in pigs, Gieling, Nordquist, and van der Staay (2011) list nothing that would fall within the field of physical cognition, as we are considering it here. The same applies to goats: They demonstrated learning, but not insight, in the solving of a two-step puzzle-box problem (Briefer, Haque, Baciadonna, & McElligott, 2014). Although most cognitive work with pigeons has been in the visual domain, there are a few studies of classic problem-solving tasks, such as obstacle removal (Nakajima & Sato, 1993) and using a box to reach an inaccessible object (Cook & Fowler, 2014; Epstein, Kirshnit, Lanza, & Rubin, 1984). Although the pigeons succeeded in these tasks, the authors conclude that their performance could be accounted for by straightforward operant conditioning processes, and did not involve insight into the structure of the problem.

To summarize, therefore, physical cognition is not a domain in which dogs excel, and their performance is at least equaled by other members of at least two of our three comparison groups.

### Spatial cognition

In considering spatial cognition, we need to distinguish small-scale and large-scale situations. The small scale involves an animal finding its way around within a small area, often its own home, a room in a laboratory, or at most a field—an area that the animal either knows well or can come to know well. The large scale involves navigation on the scale of kilometers, or even thousands of kilometers. It is not clear that the same cognitive capacities are required for both. It is not a hard and fast distinction: Many animals will have both a core area (definitely small scale) and a much larger home range within which techniques normally used for large-scale navigation might be appropriate. A guide dog leading its owner around their own neighborhood or town, for example, is operating at this intermediate or combined scale.

Dogs are certainly able to learn the characteristics of a small area well, as is shown by their performance in disappearing objects tests (Fiset, Gagnon, & Beaulieu, 2000), radial mazes (e.g., Macpherson & Roberts, 2010) and analog tasks (Fabrigoule, 1974), simple mazes (e.g. Fabrigoule, 1976), or matching to position tasks (e.g., Head et al., 1995). A persistent obstacle to performing well in simple detour tasks is dogs' tendency to perseverate—the inability to switch from a previously reinforced path to a new one (Osthaus, Marlow, & Ducat, 2010). In typical experimental conditions, they use spatial cues preferentially over visual patterns (Dumas, 1998), though they do use landmarks to establish routes (e.g., Fiset, 2009). Although Macpherson and Roberts (2010) found that dogs’ working memory for locations had quite a low capacity, their longer term memory for places can be excellent. For example, they can find their way to a designated place by a novel route (e.g., Chapuis, 1975; Fabrigoule & Sagave, 1992), even when blindfolded (Cattet & Etienne, 2004), though not without error (Seguinot, Cattet, & Benhamou, 1998); they can also remember what objects are located at a given place (Kaminski, Fischer, & Call, 2008).

On the small scale, a significant problem in dogs’ spatial behavior is their frequent inability to detour, especially at close range, as already noted by Köhler (1925, p. 27). Dogs’ problems with this task, with the more complex string-pulling tasks, and some other physical cognition tasks can be traced to proximity error—the capture of attention, and behavior, by a nearby reward, which actually has to be obtained by moving away from it, or at any rate not directly towards it (Osthaus, Lea, & Slater, 2003, 2005). They can learn to avoid the proximity error, however, either by relying more on external cues (e.g., Fiset, Beaulieu, LeBlanc, & Dube, 2007) or by observing a human making a successful detour (Pongrácz et al., 2001).

Reports of large-scale navigation by dogs, though not uncommon in the lay media, are largely anecdotal, and do not offer much more than can be found in Romanes (1886, Chapter 16). It has been claimed that dogs show systematic orientation when defecating, and that this is mediated by a magnetic sense (Hart et al., 2013), but there is no direct evidence of the use of such a sense in navigation. There is no evidence for a difference in large-scale navigational abilities between dogs and wolves. In the wild, wolves can have very large home ranges, of 100 km^2^ and above (Benson & Patterson, 2015), and finding their way around these would require mechanisms that would allow navigation over tens of kilometers. However, feral dogs have been observed to inhabit home ranges that are almost as large (up to 70 km^2^: Gipson, 1983), and, given the same local conditions, wolves and dogs exhibit the same optimized resource utilization (Boitani & Ciucci, 1995).

Spatial cognition has not been studied in such detail in any other carnivorans, though several groups, particularly pinnipeds, range very widely or migrate seasonally, or both, so they must be capable of accurate long-distance navigation. Spotted hyenas have territories that can reach up to 320 km^2^, varying in size with the seasons (Trinkel, Fleischmann, Steindorfer, & Kastberger, 2004), so like wolves and dogs they must be capable of long-range navigation. On the smaller scale, cats are good at locating hidden objects, though relying primarily on egocentric cues (Fiset & Doré, 1996); European badgers have been shown to learn simple spatial discriminations well, using landmarks (Mellgren & Roper, 1986); while American black bears were shown to have comparatively modest spatial learning ability (Zamisch & Vonk, 2012). Perdue, Snyder, Zhihe, Marr, and Maple (2011) found that giant pandas showed sex differences in spatial ability, with males showing greater ability than females, whereas Asian short-clawed otters did not; these results are in accordance with Gaulin and Fitzgerald’s (1986) range size hypothesis, since panda males have larger home ranges than females, but short-clawed otter males do not.

Among the other social hunters, dolphins are like pinnipeds in ranging widely (and some other odontocetes migrate seasonally), so they must have advanced navigational abilities. The most systematic tests of spatial ability at the medium scale, however, are in chimpanzees, which have been shown to have accurate knowledge and memory of the location of potential food sources within their (substantial) home ranges (Janmaat, Ban, & Boesch, 2013), and similar abilities were shown in captive tests (Mendes & Call, 2014)—though so far as is yet known, this ability is called upon in their frugivorous rather than their carnivorous feeding behavior.

Basic spatial learning has been investigated in most domestic animals, for example, in radial maze tests with pigs (Laughlin & Mendl, 2004), or finding food in a designated place in cows (Laca, 1998) and horses (McLean, 2004). The most detailed findings are with sheep, with many studies showing that they are highly sensitive to the distribution of different foods across a pasture, and remember it well (e.g., Dumont & Petit, 1998; Edwards, Newman, Parsons, & Krebs, 1996; Hewitson, Dumont, & Gordon, 2005). In terms of long-range navigation, however, the abilities of the homing pigeon exceed those that have been demonstrated in any other domestic species; the literature is too extensive to be reviewed here, and too well-known to need review. The point that does need to be made, however, is that although the perceptual abilities used in homing are presumably ancient, the use of them for long-distance navigation apparently developed in domestication: Although the ancestral rock dove may range over several kilometers for foraging purposes (Baldaccini, Giunchi, Mongini, & Ragionieri, 2000), it does not migrate over long distances.

In summary, the literature on spatial cognition is unsatisfactory from a comparative point of view. Few direct comparisons can be made between species working on the same standardized tests, and we often find ourselves inferring spatial ability from a species’ ecology. Dogs have certainly shown good performance in spatial tasks, but the same is true of other species in all our comparison groups, and we have no evidence that they stand out as exceptional in this domain.

### Social cognition

Social cognition has been the focus of much of the recent research on dogs and wolves, so the literature on it is extensive. However, the results are relatively familiar, and we therefore deal with them briefly here, citing examples rather than providing an exhaustive guide to the literature. We consider three aspects of social cognition: using another animal’s behavior as a cue for an arbitrary response; social learning, that is, learning an adaptive behavior as a result of observing another animal behaving in the same way; and “theory of mind,” that is, responding in a way that suggests an understanding of another animal’s cognitive processes. Because of the volume of literature available, we will summarize our comparative conclusions under each of these subheadings.

#### Using another animal as a cue

The simplest of all kinds of social cognition is using the presence, nature, or behavior of another animal, whether conspecific or allospecific, as a cue in a learned task or in solving a problem. This has been the focus of an enormous amount of recent research in dogs, particularly in relation to dogs’ use of points or gaze from humans. One major reason for this research focus is that dogs do seem to be highly sensitive to such signals. A second major reason has been the “domestication hypothesis”: the suggestion that domestication has selected for particular sensitivity to human cues in dogs (Hare & Tomasello, 2005). In consequence there has been considerable effort to use pointing or gaze tasks to compare dogs with wolves. In order to test alternative, ontogenetic explanations of dogs’ social skills, there has also been much effort to compare groups of dogs whose life has involved different kinds or degrees of interaction with humans. Much of this literature is reviewed by Kaminski and Nitzschner (2013), though an earlier review by Miklósi and Soproni (2006) is also highly relevant because it focuses on comparative questions.

To summarize this extensive literature, we can say that since the original formal demonstration of dogs’ use of human pointing and gaze by Miklósi, Polgárdi, Topál, and Csányi (1998), the phenomenon has been thoroughly explored and its limitations more or less determined. Dogs can follow a variety of different kinds of human point (for review, see Miklósi & Soproni, 2006), and some studies suggest that both dogs and wolves can follow human gaze even around opaque barriers (Met, Miklósi, & Lakatos, 2014; Range & Virányi, 2011), though earlier studies failed to find evidence for this in dogs (Agnetta, Hare, & Tomasello, 2000). It seems likely that response to point and gaze use emerge very early in dog development (Gácsi, Kara, Belényi, Topál & Miklósi, 2009; Riedel, Schumann, Kaminski, Call, & Tomasello, 2008; Zaine, Domeniconi, & Wynne, 2015). The actual utilization of these cues is somewhat different in dogs, or wolves, that have had less interaction with humans (D’Aniello et al., 2017; Udell, Dorey, & Wynne, 2010; Virányi et al., 2008). It also differs between wolves and dogs, but the exact nature of these differences remains controversial (Miklósi et al., 2003; Udell, Dorey, & Wynne, 2008); although attention to human points emerges later in hand-reared wolf cubs than in hand-reared puppies, with appropriate training it can reach the same level in both groups (Virányi et al., 2008).

There is some evidence of what might be thought of as the complementary phenomenon, dogs themselves performing something corresponding to pointing. Some hunting dogs are selectively bred for their tendency to point at fallen game (Parra, Méndez, Cañón, & Dunner, 2008), though so far as we know the accuracy with which they do so has not been investigated, and there is no evidence that the communication involved serves any function for the dog. In experimental situations, dogs used gaze alternation to draw their owners’ attention to the location of a hidden toy (Marshall-Pescini, Colombo, Passalacqua, Merola, & Prato-Previde, 2013; Miklósi, Polgárdi, Topál, & Csányi, 2000; Persson, Roth, Johnsson, Wright, & Jensen, 2015), and few of the dogs involved in these experiments were from pointing breeds. Dogs can be shown to literally look to their owners or handlers for help when faced with an unsolvable task (Marshall-Pescini, Rao, Virányi, & Range, 2017), but this is true also for wolves, and for both dogs and wolves seems to occur regardless of the how closely they have lived with humans. Both pack-living dogs and wolves follow their conspecifics’ gaze (Werhahn, Virányi, Barrera, Sommese, & Range, 2016). Giving a battery of different cognitive tasks to both dogs and chimpanzees, MacLean, Herrmann, Suchindran, and Hare (2017) found that individual differences in dogs’ social skills at a range of cooperative communicative problems were correlated in dogs (as they are in human infants), but not in chimpanzees. This might indicate a convergent evolution between dogs and humans of factors underlying social cognition, probably caused in dogs by artificial selection, and not necessarily cognitive in nature: MacLean et al. suggest that enhanced social tolerance and reduced aggression might be involved.

Beyond the experiments with dogs and wolves noted above, there is limited literature on pointing or gaze-following in other carnivorans. In Miklósi and Soproni’s (2006) review, two aquatic carnivoran species had the highest rate of success in spontaneous use of human points of all taxa (bottlenose dolphins, Pack & Herman, 2004, and see also Xitco, Gory, & Kuczaj, 2001; and a grey seal, Shapiro, Janik, & Slater, 2003). A recent report of successful use of points by Californian sea lions continues that trend (Malassis & Delfour, 2015). Among the social hunters, there is much more evidence. The literature on gaze following in chimpanzees is vast, and their ability to use it (including around barriers: Braeuer, Call, & Tomasello, 2005) is not doubted (for a recent overview, see Itakura, Das, & Farshid, 2017). However, in Miklósi and Soproni’s review, apes’ comprehension in a range of pointing situations emerged as worse than that of dogs, and in a direct comparison, using imperative pointing, Kirchhofer, Zimmermann, Kaminski, and Tomasello (2012) confirmed that they did more poorly than dogs. Miklósi and Soproni raise the possibility that the superiority of dogs over chimpanzees arises because the majority of pointing situations are inherently cooperative, and chimpanzees do better in competitive situations. Among domestic animals, evidence is more limited, but goats are able to use conspecifics’ gaze to find food (Kaminski, Riedel, Call, & Tomasello, 2005), and young pigs can make use of some human pointing gestures (Nawroth, Ebersbach, & Von Borell, 2014).

To sum up, therefore, dogs’ ability to use other animals’ behavior as a cue is impressive, but not unique; other carnivorans are better at these tasks. No other social hunter (apart from the wolf), however, has been shown to do as well as dogs. However, other domestic species may do as well as dogs.

#### Social learning

Beyond using another animal as a cue, the next form of social cognition to be considered is the use of another animal as an aid to learning a task—what is generally referred to as social learning, where an observer animal learns a new behavior more quickly as a result of witnessing a demonstrator perform it. Within this field, it is standard practice to distinguish local or stimulus enhancement (the observer is attracted to an appropriate place or object by the demonstrator’s attention to it), emulation (a term that has been used in different ways, but most often meaning that the observer’s relevant motivation is raised by seeing the demonstrator perform a motivated behavior), and motor imitation (the observer becomes more likely to make a particular response as a result of seeing the demonstrator perform it). Whiten, Horner, Litchfield, and Marshall-Pescini (2004) give a detailed overview of these concepts. All have been documented in dogs (local enhancement, e.g., Mersmann, Tomasello, Call, Kaminski, & Taborsky, 2011; emulation, e.g.. Miller, Rayburn-Reeves, & Zentall, 2009a; motor imitation, e.g., Huber et al., 2009).

An interesting special case of motor imitation is “overimitation,” where an observer repeats a demonstrator’s actions, including responses that are not necessary to achieve the goal; the opposite, the omission of unnecessary actions, has been called “rational imitation,” because it can be argued to demonstrate a fuller understanding of the situation (Gergely, Bekkering, & Kiraly, 2002). It is currently disputed whether dogs show overimitation. A. M. Johnston, Holden, and Santos (2017) showed that when imitating human actions, both dogs and dingoes, unlike human infants, showed a decreasing tendency to include unnecessary actions. The authors argue that this tendency does not mean that the canids demonstrated a superior understanding of the procedure, but rather that it casts doubt on the extent to which dogs are in fact imitating the actions of human models, rather than using them as a cue. However, about 70% of A. M. Johnston et al.’s dogs did show overimitation initially, and Huber, Popovová, Riener, Salobir, and Cimarelli (2018) have brought forward further evidence for canine overimitation.

While it is clear that dogs can learn from the behavior of other dogs, in the current state of research we still cannot say whether or not this means that they understand the goals of the demonstrator, as Huber, Range, and Virányi (2014) point out. The same applies to the literature on the “Do as I Do” paradigm, an extension of social learning that requires the acquisition of what might be called an “imitation set.” The animal is trained to repeat actions by the demonstrator on a specific signal (e.g., Fugazza & Miklόsi, 2014, 2015; Topál et al., 2006). In the training stage of these experiments, the actions involved had all been trained previously, by operant conditioning over many trials: The dogs were thus selecting between known actions on the basis of the human demonstrator’s behavior, and it is not necessarily the case that they recognized the similarity between the demonstrator’s behavior and their own. The important question is what happens when wholly novel responses are demonstrated. Topál et al. (2006), using a highly trained assistance dog, state that there were “significant limitations in [the dog’s] imitative abilities” (p. 355). The dog performed sufficiently well when the new actions were comparable to the trained ones, such as transporting an object from A to B, but faltered when completely new behaviors were required. Fugazza and Miklόsi (2014, 2015) provided stronger evidence that the Do as I Do procedure facilitates learning of novel complex actions or sequences of actions, but they do not claim that the novel tasks were ever acquired immediately, as should follow if a true imitation set has been acquired. It would be difficult to be certain that acquisition is immediate, and we suggest that a new kind of control procedure might be helpful in Do as I Do experiments: Acquisition of novel responses following Do as I Do training could be followed with testing in which the required response was signaled by the trainer either making that response, or a different response. If the truly imitative response was acquired faster than a pseudo-imitative response, the evidence that the dogs recognized the similarity between the demonstrator’s behavior and their own would be stronger.

What is the evidence for social learning in our comparison groups? As regards other carnivorans, the most direct comparison is a study of Range and Virányi (2014), who found that young wolves were better at a motor-imitation task than were dogs of the same age and rearing conditions. Benson-Amram, Heinen, Gessner, Weldele, and Holekamp (2014) tested social learning skills in spotted hyenas and found only weak evidence of it, with local or stimulus enhancement as the most likely explanation. Borrego and Dowling (2016) reported that lions that had previously failed to solve a physical problem succeeded when in the company of a lion that had previously solved it. The social transmission of knowledge about feeding grounds, seen, for example, in sea lions (Schakner et al., 2017) can be considered a case of local enhancement. When it comes to noncarnivoran social hunters, however, there is copious evidence of imitation in both chimpanzees and bottlenose dolphins, and in these species it does seem to be a specifically social skill. For example, chimpanzees can acquire tool use by observation of others, and this seems to depend on witnessing an animate other using the tool (Hopper, Lambeth, Schapiro, & Whiten, 2015). Furthermore, Do as I Do responding can be established: Hayes and Hayes (1952) used it in their attempt to teach the chimpanzee Vicki human speech, and while they did not succeed in getting her to copy speech sounds (probably because of anatomical limitations), she readily imitated physical actions. In a review of the literature on dolphin imitation, Herman (2002) concluded that they have a generalized and highly flexible capacity for imitative acts. Some domestic animals also show a tendency to social learning: Goats have shown social learning from humans in detour tasks (Nawroth, Baciadonna, & McElligott, 2016), and pigs are at least able to use the behavior of others to help them find food more quickly (Held, Mendl, Devereux, & Byrne, 2000). Both of these fit the definition of local enhancement. Horses, however, did not profit from seeing a conspecific take a detour (Rorvang, Ahrendt, & Christensen, 2015).

In summary, it is clear that dogs have impressive capacities for social learning. As far as current evidence can tell us, they seem to do better at these tasks than any other carnivorans apart than wolves. However, some social hunters, particularly dolphins and chimpanzees, have shown clearer evidence of motor imitation than have dogs. Other domestic animals have not yet been shown to have social learning capacities beyond local or stimulus enhancement, and horses have failed even at that level.

#### Theory of mind

More elaborate forms of social cognition are generally tied, in one way or another, to the concept of “theory of mind,” introduced into animal cognition by Premack and Woodruff (1978). Three lines of investigation have been pursued in the search for evidence that animals have some understanding of other individuals’ minds. Can an animal understand what another animal can perceive, and predict what it will understand (perspective taking)? And if so, can it use that information, either to mislead the other animal (deception) or to enter into the same state of mind (empathy)?

There is some evidence for perspective taking in dogs. They frequently react appropriately to what a human can or cannot know about a situation (Catala, Mang, Wallis, & Huber, 2017; Kaminski, Bräuer, Call, & Tomasello, 2009; Maginnity & Grace, 2014), though this ability is not seen in all dogs (Udell, Dorey, & Wynne, 2011). With regard to deception, dogs can also learn to respond differently to humans who habitually deceive them about the location of food, compared with truthful humans (Petter, Musolino, Roberts, & Cole, 2009), though this is effectively an operant discrimination task rather than requiring any special social cognition. Dogs themselves may engage in deception in play (Mitchell & Thompson, 1993), and they can learn to lead a human who will not share food with them away from the food source (Heberlein, Manser, & Turner, 2017), though this result can again be interpreted as differential conditioning, without the necessity of possessing a theory of mind.

The question of whether dogs display empathy has largely been investigated in relation to humans rather than to conspecifics, and the answer is moot. There have been a series of investigations of contagious yawning, a behavior often taken to indicate empathy, and it is clear that dogs do show this phenomenon. While the interpretation of the data is still controversial, at least the latest studies (Romero, Konno, & Hasegawa, 2013; Silva, Bessa, & de Sousa, 2012) strongly suggest the involvement of social motivation. Another simple form of empathy is emotional contagion, in which signs of emotion in one individual lead to the expression of the same emotion in an observer. Dogs can discriminate human emotional expressions in a preferential looking task (Albuquerque et al., 2016), and Huber, Barber, Faragó, Müller, and Huber (2017) claim that they do express the corresponding emotion when they perceive an emotional reaction in humans. More generally, Custance and Mayer (2012) showed that dogs tend to show submissive behavior toward people showing visible distress, which at least suggests emotional contagion. It might also suggest a desire to comfort, which links this study to a further approach to empathy, the idea that dogs would seek help for a person in distress or danger. Macpherson and Roberts (2006) found no evidence that they would. Bräuer, Schönefeld, and Call (2013) claimed to show that dogs would help humans if the situation was clear enough (in their case, opening a door), but, unfortunately, their experiment lacks a control condition (an alternative highly trained, but irrelevant behavior) and the “helping behavior” can again be explained by differential conditioning. Piotti and Kaminski (2016) found no clear evidence that dogs engaged selectively in “helpful communication” when given a choice between looking toward two targets, one “relevant” to a human and the other not. Also, their definition of “relevant” overlapped with “previously handled by” the experimenter. The assumption that a human holding a pen is perceived by a dog as in need or want of a writing pad first needs to be established before this can be used to test for “helpful behavior.”

Both perspective taking and empathy should, in principle, help animals solve problems that require cooperation. Two recent studies have reported some degree of cooperation in problem solving by dogs, in a door-sliding task (Bräuer, Bos, Call, & Tomasello, 2013) and the two-rope task that has been widely used with other species (Ostojić & Clayton, 2014); and Range and Virányi (2014) have argued that cooperativeness is, in fact, an inherent quality of wolves’ social lives, and contributed greatly to their successful domestication as dogs.

Turning to comparisons, some wolves, like some dogs, show evidence of perspective taking (Udell et al., 2011), but we know of no evidence for other carnivorans. Early role-reversal tasks suggested that chimpanzees are capable of perspective taking (Povinelli, Nelson, & Boysen, 1992), and although this conclusion was initially called into question by experiments using the Guesser–Knower paradigm (e.g., Povinelli & Eddy, 1996), it has subsequently been confirmed in several experiments using competitive rather than cooperative tasks (e.g., Bräuer, Call, & Tomasello, 2007; Kaminski, Call, & Tomasello, 2008). Investigation of so-called Level-2 perspective taking, however, suggests that chimpanzees do not truly understand another’s mistaken perspective, in the way that children do (Karg, Zchmelz, Call, & Tomasello, 2016). Among domestic animals, both goats (Nawroth, Brett, & McElligott, 2016) and horses (Malavasi & Huber, 2016) demonstrate the same kinds of intentional and referential communication with humans as dogs do. Pigs show some signs of perspective taking (Held, Mendl, Devereux, & Byrne, 2001), though the authors of that study are cautious as to whether their results imply theory of mind.

As regards deception, again we know of no evidence for other carnivorans. Despite early claims (Woodruff & Premack, 1979), the experimental evidence that chimpanzees engage in deception is weak; however, they do seem to conceal food from potential rivals (Hare, Call, & Tomasello, 2006; Osvath & Karvonen, 2012), behavior which also reflects future planning, discussed further below. The deceptive use of social signals, which has been the subject of much discussion in primates in general, has been reported for chimpanzees in natural situations (e.g., Slocombe & Zuberbühler, 2007). But there is no evidence of deception in any of the other social hunters we have considered, or in noncarnivoran domestic species.

Similarly, we have found no claims for empathy in carnivorans, except that Romero, Ito, Saito, and Hasegawa (2014) have observed contagious yawning in wolves. However, there is copious and longstanding evidence for it in chimpanzees, both from formal experimental tasks and in more natural social situations (see the review by Clay, Palagi, & de Waal, 2018); it has not, apparently, been studied in dolphins. Spontaneous helping behavior has also been reported in chimpanzees (Greenberg, Hamann, Warneken, & Tomasello, 2010); it is widely claimed anecdotally in dolphins, but formal demonstrations are lacking.

Cooperation has been demonstrated in spotted hyena problem solving (Drea & Carter, 2009), but the only report in any other carnivoran is of modest cooperative problem-solving abilities in two species of otter (Schmelz, Duguid, Bohn, & Volter, 2017). It has been extensively studied in chimpanzees, and is clearly something they are capable of, though only if the social conditions are right (e.g., Melis, Hare, & Tomasello, 2006). Dolphins similarly often appear to be cooperating in natural situations, but formal experiments do not always find successful cooperation. A recent report using an analogue of the two-rope task (Kuczaj, Winship, & Eskelinen, 2015) is more promising, but its methodology has been heavily criticized (King, Allen, Connor, & Jaakkola, 2016). Once again, we have found no formal studies of cooperation in domestic animals other than dogs: indeed, as regards pigeons, Boakes and Gaertner (1977) used an experiment that appeared to demonstrate cooperation to show that an early experiment on dolphin cooperation (Bastian, 1967) could be accounted for by simple conditioning.

In summary, the social use of theory of mind is an area where we have too little comparative data to draw firm conclusions: It is only really in dogs and chimpanzees that extensive experimentation has been carried out, though cooperative problem solving is now beginning to be studied in a wider range of species. In experiments carried out so far, chimpanzees are less likely than dogs to solve tasks requiring perspective taking or deception and more likely to show evidence of empathy, but the type of tasks in which they do either seem to be different. What little other comparative evidence we have suggests that dogs and wolves may do better in such tasks than other domestic animals, but this conclusion can only be tentative.

### Self-consciousness and mental time travel

Linked to the question of theory of mind is the possibility of self-consciousness. The standard way of examining this in animals is the mirror-mark test, pioneered by Gallup (1970) for use with chimpanzees. We know of no evidence that dogs respond to their image in a mirror, following marking, in the same way as chimpanzees (and humans). Gatti (2016) has argued that an alternative way of approaching the problem is through dogs’ own urine marking. His study provided evidence of a kind of self-recognition, in that the dogs reacted differently to their own than to others’ smells; but this is different from the recognition of an animal’s own body, which is shown in the mirror-mark test, and in any case, self-recognition, even mirror self-recognition, though perhaps a necessary condition for ascribing an animal (or a person) self-consciousness, is not a sufficient condition (Morin, 2011).

A different way of approaching the question of an animal’s awareness of self is through the possibility of what Suddendorf and Corballis (1997) call “mental time travel”—the ability to project oneself into past events, through episodic memory, or into future events, through episodic future thought, or planning. Tulving (1972), who introduced the term *episodic memory*, subsequently argued that these abilities require “autonoetic consciousness,” that is, knowledge of the self (Tulving, 1985). Although many authors have claimed to show an analogue of human episodic memory in other animals, using the “what-where-when” test introduced by Clayton and Dickinson (1998), comparable studies with dogs have only shown an association of “what” and “where” that does not qualify as a full analogue (Fujita, Morisaki, Takaoka, Maeda & Hori, 2012; Kaminski et al., 2008). However, Zentall, Clement, Bhatt, and Allen (2001) introduced an alternative approach to episodic memory, in which animals are given an unexpected test requiring memory of their own behavior. Fugazza, Pogány, and Miklósi (2016) adapted this procedure for use with dogs, but instead of testing the subjects for memory of their own behavior, they gave them an unexpected test for memory of the behavior of the demonstrator in a Do as I Do experiment. The dogs’ tendency to respond correctly declined quite rapidly over time, and Fugazza et al. argue that this shows evidence of episodic memory.

The mirror-mark test was first developed for chimpanzees, and although it does not work with every individual, and there has been controversy over its interpretation, the basic result of attention to the affected body part seems to be well established (Gallup et al., 1995; Heyes, 1994). And despite the difficulties of carrying out parallel studies with an animal that cannot use its limbs to touch most parts of its body, there are several demonstrations that dolphins, too, respond by inspection to a mark placed on their body (Marten & Psarakos, 1995; Reiss & Marino, 2001). There are no comparable reports for other carnivorans, nor for noncarnivoran domestic species.

We know of no laboratory studies of episodic memory or planning in carnivorans other than dogs and wolves. The opportunity for such studies clearly exists, because a number of carnivores show scatter hoarding, which was used as a vehicle for the earliest studies of animal episodic memory: Macdonald (1976) lists the earlier evidence across a wide range of carnivorans. Caching has been well studied in both red and Arctic foxes (Careau, Giroux, & Berteaux, 2007; Macdonald, 1976; Sklepkovych & Montevecchi, 1996), but it is not confined to them; for example, Way and Cabral (2009) describe it in coyote-wolf hybrids. Although scatter hoarding clearly places demands on an animal’s memory, the only attempt to use the behavior to study cognition seems to be a field study of apparently planful caching in a South American mustelid, the tayra (Soley & Alvarado-Díaz, 2011).

Surprisingly, we have found no direct studies of episodic-like memory in chimpanzees or dolphins, but there has been a spate of investigations of the other type of mental time travel, planning, and intention in chimpanzees. For example, in the laboratory, chimpanzees will produce tools for future use (Bräuer & Call, 2015). They will also direct their travels with apparent forethought within a computerized maze (Beran, Parrish, Futch, Evans, & Perdue, 2015) or in their home range in the wild (Ban, Boesch, & Janmaat, 2014; Janmaat, Polansky, Ban, & Boesch, 2014). Again, the interpretation of these results remains controversial (Osvath & Osvath, 2008; Suddendorf, Corballis, & Collier-Baker, 2009), but there seems little doubt that chimpanzees show a kind of future-oriented behavior that has not been demonstrated in dogs or, indeed, in our other comparison species.

Among the noncarnivoran domestic species, pigs have been reported to show episodic-like memory in a modified what-where-when test (Kouwenberg, Walsh, Morgan & Martin, 2009), and Fuhrer and Gygax (2017) argue that their results on time estimation in pigs provide supporting evidence. Zentall et al. (2001) argue that their unexpected test experiment provides evidence for episodic-like memory in pigeons.

In summary, studies designed to look for evidence of self-consciousness in dogs have not yet found much positive evidence. The same is true of other carnivorans and other domestic animals, although both in dogs and in some domestic animals there is evidence for episodic-like memory. However, the only animals that reliably do well in tests of self-consciousness are two social hunters: chimpanzees and dolphins.

## The comparative intelligence of dogs

In this section, we gather up the comparative summaries that we have made throughout the previous section in order to review how dogs’ success at cognitive tasks compares, across domains, with that of other carnivorans, other social hunters, and other domestic animals.

- In associative learning, we found no evidence that associative learning is in any way unusual in dogs.
- In perception and sensory cognition, the situation is more nuanced. Dogs’ olfactory abilities are excellent, but similar abilities have been found in some other carnivorans and domestic animals. Dogs’ sensory cognition seems to be similar to that of other carnivorans and social hunters that have been tested, and some but not all domestic animals.
- Physical cognition is not a domain in which dogs excel, and their performance is at least equaled by other members of all three of our comparison groups.
- In spatial tasks, dogs have shown good performance, but the same is true of other species in all our comparison groups, and we have no evidence that they stand out as exceptional in this domain.
- Social cognition is the domain in which we have most information. Dogs have an impressive ability to use other animals’ behavior (particularly the behavior of humans) as a cue. However, some other carnivorans are even better at these tasks, and some other domestic species may do as well as dogs, though no other social hunters (except for wolves) have been shown to do as well. Dogs also have impressive capacities for social learning, and they seem to do better at these tasks than any other carnivorans, except wolves. Qualitatively speaking, they have not demonstrated any capacities that have not also been shown in other social hunters, and dolphins and chimpanzees show clearer evidence of motor imitation. Dogs perform as well as or better than other domestic animals on social learning tests. As regards tests inspired by theory-of-mind considerations (perspective taking, deception, and empathy), we have too little comparative data to draw many conclusions. In experiments carried out so far, chimpanzees are more likely than dogs to solve tasks requiring perspective taking, though the evidence base for dogs’ perspective taking is improving, and dogs may do better than chimpanzees in cooperative situations. Chimpanzees are more likely than dogs to show evidence of deception or empathy. What little other comparative evidence we have suggests that dogs and wolves may do better in such tasks than other carnivorans and domestic animals, but this conclusion can only be tentative.
- Except for a claim of episodic-like memory, we have no firm evidence of self-consciousness in dogs, either from analogues of the Gallop mark test or from tests of mental time travel. The same is true of other carnivorans and domestic animals, but two social hunters, chimpanzees and dolphins, have reliably shown such evidence.

What can we draw out of these summaries of the evidence? Accepting always that many desirable comparisons have not yet been made, and that trying to test the same cognitive capacity in different species is fraught with methodological difficulties, we can at least draw some interim conclusions.

First, except for some (sometimes contested) details of the way dogs use the behavior of humans as cues, we have found no evidence of substantial differences in cognition between dogs in general and wolves. Dogs reared under particular circumstances may show inferior performance, and wolves may do less well in some tests requiring close attention to humans, but these differences are slight compared with those between dogs or wolves and other species. This is a particularly important comparison, both because of the phylogenetic closeness of dogs and wolves in itself, and because that closeness makes it easier to carry out truly comparable tests on the two species. Cognitively speaking, dogs are not exactly wolves, but on the evidence currently on hand, they are closer to wolves than to any other species.

Second, across the different domains of cognition, no clear pattern emerges of dogs performing more like other carnivorans, more like other social hunters, or more like other domestic animals. We conclude that the cognition of dogs is not to be understood by regarding them as essentially members of any one of these three groups: rather, they exist at the intersection of all three, as we suggested in Fig. 1.

Third, if we compare our three comparison groups, there is no pattern suggesting qualitative differences between them. In some cognitive domains, all perform more or less equally. In others, members of one group do better—for example, the use of another animal of a cue seems to be best developed among carnivorans, whereas in tests of self-consciousness, the most convincing evidence comes from two kinds of social hunters, chimpanzees and dolphins.

Finally, we do not find any evidence that dogs are exceptional among those groups, except by virtue of belonging to the other two groups. That is to say, for example, that although they differ in some ways from other carnivorans, those differences can be understood by taking into account that they are also social hunters (or recently descended from social hunters), and also domestic animals. We have unfortunately too few data on cognition in any of the canids closely related to dogs, or on the other clearly identifiable social hunters among the carnivorans (the African wild dog and the spotted hyena), to draw strong generalizations about their performance relative to dogs. However, from what we do know—mainly about spotted hyenas—there is no reason to think that their performance is worse than that of dogs, and in some cases (e.g., in tests of physical cognition) it seems to be better.

Looking at the carnivorans more widely, dogs have clearly been subjected to more cognitive tests than any other carnivoran. This makes phylogenetic comparisons difficult. It would be particularly useful to have more data on cat cognition, because cats share a long history of domestication with dogs, and also like dogs are often kept as companions or aesthetic objects rather than for use or food; but the days are long gone when we could make a point-for-point comparison of dog and cat cognition, with roughly equal amounts of data on each, as Doré and Goulet (1992) did. There are clearly some tasks, particularly in the area of physical cognition including tool use, where even the scant data we have suggest that there are other carnivorans who succeed better than dogs. There are also specialized natural tasks, such as long-range navigation and scatter hoarding, for which some other carnivorans are cognitively equipped while, so far as we yet know, dogs are not.

Considering the social hunters, again it does not appear that dogs are exceptional. As noted above, among the noncarnivoran social hunters we find two taxonomic groups, the chimpanzees and bonobos, and the bottlenose dolphins, whose cognitive capacities are clearly superior to those of dogs on some tests (e.g., in self-consciousness); and although dogs do better than chimpanzees at others (e.g., using the behavior of other animal, especially a human, as a cue), this ability seems to be widespread among carnivorans, and some other carnivorans perform better in these tasks than dogs.

Although there is a growing literature on domestic animal cognition, it contains few reports of capacities superior to those shown by dogs, though both goats and pigs at least approach many of the abilities of dogs and may turn out to match them. The detailed work that has been done on face recognition in sheep, and the wider literature on pattern recognition in pigeons, may well demonstrate superior capacities to those of dogs, but we do not yet have as detailed an account of visual cognition in dogs to compare it with. The one outstanding example is in navigation, where pigeons’ homing capacities (presumably developed in the domestic context) far exceed anything that we have experimental evidence for in dogs. It is unfortunate that we have relatively little formal knowledge so far of cognition in domestic equids or tamed raptors, because these are two groups that, like dogs, have been kept to work cooperatively with humans, and it has been argued (e.g., Kaminski & Nitzschner, 2013; Range & Virányi, 2014) that it is this aspect of dog domestication that has led to the alleged emergence of distinctive cognitive capacities. Neither the horse nor the raptor case offers an exact parallel to the way dogs have been bred and used, but in both cases, as with dogs, there is a great deal of practitioner knowledge that has been documented over a long period. As this becomes integrated with scientific study, these lines of comparison should become more fruitful.

Throughout this paper, we have been comparing dogs with other carnivorans, other social hunters, and other domestic animals. The implication is that the answer to Coppinger and Coppinger’s (2016) question, “What is a dog?” is precisely that it is an animal that belongs to all three of those groups; and all three of those qualities contribute to the cognitive position of dogs. On this basis, one might argue that one should compare social hunters with solitary hunters or herbivores, and domestic with wild species, in other taxa, to see whether those two factors add to the phylogenetic variations in cognition that seem obvious when we compare dogs with apes in general, or cetaceans in general (for example). If a gorilla or an orangutan’s cognition excels that of a dog on just the same tasks as a chimpanzee’s does, that would suggest that phylogenetic factors trump ecological ones in determining the nature of a species’ cognitive capacities. There is, indeed, no doubt that a fuller analysis like that needs to be done. There are some particularly interesting cases where we do not yet have data that we could compare with results from dogs, for example, the African painted dog (both a carnivoran and a social hunter) and the equids (domestic animals that are trained to work cooperatively with humans). However, we think that we can see, from the limited analysis so far, the direction in which it is likely to lead. Dog cognition looks quite a lot like that of other carnivorans, especially other closely related carnivorans; but it also looks somewhat like that of unrelated social hunters, and at least some unrelated domestic animals.

In the present state of our knowledge, we are led to a simple conclusion: When a broad-enough set of comparison species is considered, there is no current case for canine exceptionalism. Dog cognition is, no doubt, unique, because the cognition of every species is unique. Dogs exist at a particular intersection of phylogenetic, ecological, and anthropogenic circumstances (see Fig. 1). But on the basis of the evidence we have reviewed here, those circumstances are sufficient to account for the nature of dog cognition: It is what we would expect of cognition in a domesticated socially hunting carnivoran.

## The contribution of studies on dogs to our knowledge of comparative cognition

So, finally, what have the extensive studies of dog cognition of the past two decades brought to comparative cognition in general? We argue that there have been several major contributions. Dog cognition may not be exceptional, but dogs are certainly exceptional cognitive research subjects. There have some often rehearsed practical advantages: dogs are available in much larger numbers than any interesting comparator species except cats and horses; they do not have to be studied in captivity, or kept in laboratories, so the costs of studying them are much lower than for most of the other species we have mentioned; they have been selected for, over the millennia, based on their performance in a range of cognitively interesting tasks; and they were selected for their motivation to cooperate with humans. The systematic breeding of dogs for appearance instead of behavior only started in the 19th century: The first recorded dog show took place in 1859 (Rooney & Sargan, 2008).

These are essentially methodological points, but there are more substantive contributions as well. Our knowledge of nonhumans’ understanding of pointing, gaze, and other human signals has been greatly expanded through studies on dogs. The same literature has led to informed theorizing about cognitive aspects of the domestication process (e.g., Hare & Tomasello, 2005), largely ignored in previous accounts of dog domestication, even relatively recent ones (e.g., Clutton-Brock, 1995). There are several fields of cognition—empathy, for example—where almost our only nonprimate evidence comes from dogs, and the number of these seems likely to grow because the cooperativeness of dogs means that more complex research designs can be carried through than could be contemplated with less obliging subjects (e.g., cats). And although dogs may not be typical carnivorans, or typical social hunters, or typical domestic animals, what we know about cognition in all those groups consists to a substantial extent of what we know about dog cognition.

A comparative approach seems like the antithesis of the “model organism” approach to biology. The comparative approach recognizes that there is no such thing as a generalized animal, only particular animals—that, indeed, is the reason we gave at the beginning of this paper for comparing dogs with a principled selection of species, rather than other animals in general. Nonetheless, it is clear that we cannot explore every species’ cognition in detail, any more than we can compare dog cognition with that of every other species. We have to understand the cognition in a few species really well, and then we can use that understanding as a framework to design investigation of cognition of other species as they become of interest. The flowering of work on dog cognition this century has placed dogs squarely within the small set of species whose cognition we can claim to understand reasonably well. It is a highly valuable addition because as the unique combination as a carnivoran, a social hunter, and a domestic animal, it is unlike the other species whose cognition has been investigated extensively. As scientists whose interest is essentially in comparative cognition, we hope that we can now begin to use our knowledge of dog cognition to go beyond the study of dogs and look at more of the comparator species. And, of course, in doing so, we will also expand our understanding of what, fundamentally, a dog uniquely is.
